# Marked Campylobacteriosis Decline after Interventions Aimed at Poultry, New Zealand

**DOI:** 10.3201/eid1706.101272

**Published:** 2011-06

**Authors:** Ann Sears, Michael G. Baker, Nick Wilson, Jonathan Marshall, Petra Muellner, Donald M. Campbell, Robin J. Lake, Nigel P. French

**Affiliations:** Author affiliations: University of Otago, Wellington, New Zealand (A. Sears, M.G. Baker, N. Wilson);; Massey University, Palmerston North, New Zealand (J. Marshall, P. Muellner, N.P. French);; New Zealand Food Safety Authority, Wellington (D.M. Campbell);; Institute of Environmental Science and Research Ltd, Christchurch, New Zealand (R.J. Lake)

**Keywords:** bacteria, foodborne infections, Campylobacter, epidemiology, surveillance, poultry, food supply, bacterial typing, research, New Zealand

## Abstract

Beginning in the 1980s, New Zealand experienced rising annual rates of campylobacteriosis that peaked in 2006. We analyzed notification, hospitalization, and other data to explore the 2007–2008 drop in campylobacteriosis incidence. Source attribution techniques based on genotyping of *Campylobacter* *jejuni* isolates from patients and environmental sources were also used to examine the decline. In 2008, the annual campylobacteriosis notification rate was 161.5/100,000 population, representing a 54% decline compared with the average annual rate of 353.8/100,000 for 2002–2006. A similar decline was seen for hospitalizations. Source attribution findings demonstrated a 74% (95% credible interval 49%–94%) reduction in the number of cases attributed to poultry. These reductions coincided with the introduction of a range of voluntary and regulatory interventions to reduce *Campylobacter* spp. contamination of poultry. The apparent success of these interventions may inform approaches other countries could consider to help control foodborne campylobacteriosis.

## Abstract 2

A population-level food safety response successfully reduced disease incidence.

Campylobacteriosis is a common bacterial gastroenteritis reported in New Zealand and many other industrialized countries, with most cases caused by *Campylobacter jejuni* (1,2). Campylobacteriosis has been a notifiable disease in New Zealand since 1980, and medical practitioners are required to report confirmed or suspected cases to their local public health service (3). Campylobacteriosis notifications rose steadily after campylobacteriosis first became notifiable and peaked in 2006 at >380 per 100,000 population (4). A concomitant increase in campylobacteriosis hospitalizations has been noted, which suggests this rise in notifications is unlikely to be artifactual (3,5).

To help inform prevention and control strategies, research efforts have been directed at establishing the likely contributors to this rise in campylobacteriosis incidence. Consistent with international findings (6–8), New Zealand investigations implicated poultry meat as a significant source of foodborne sporadic campylobacteriosis (9–13). A relatively small case–control study in Christchurch in 1992–1993 reported several poultry-associated risk factors, including consumption of undercooked poultry (10). A larger national case–control study in 1994–1995 reported similar findings, with a combined population-attributable risk of poultry-related exposures >50% (9). A systematic review also concluded that poultry consumption was a prominent risk factor for sporadic campylobacteriosis in New Zealand (11). Reports noted the rise in campylobacteriosis was closely correlated with an increase in consumption of fresh poultry (14).

Microbiological source attribution approaches have also been used to estimate the contribution of different sources and transmission pathways of campylobacteriosis in New Zealand. These techniques involve examining the epidemiology of campylobacteriosis at the genotype level by comparing *C. jejuni* genotypes from humans with those found in a range of food and environmental sources. In 2005, a major source attribution study for campylobacteriosis was initiated at a sentinel surveillance site in the Manawatu region of New Zealand (12). *C. jejuni* isolates from cases notified in the region were genotyped by using multilocus sequence typing (MLST) and compared with isolates recovered from food and environmental sources (12,13). Statistical modeling was used to apportion human cases to potential disease sources, thereby estimating each source’s relative importance (13,15,16). This modeling revealed that >50% of sporadic campylobacteriosis cases in the region were attributable to poultry (12,13).

On the basis of these findings, public health professionals advocated for more rigorous controls on foodborne pathways of campylobacteriosis, particularly for poultry (5,14). One intervention promoted was the freezing of fresh poultry meat to reduce levels of *Campylobacter* spp. contamination, with fresh poultry allowed to be sold only when it could be shown to pose a low risk to human health (5,14). In late 2006, the New Zealand Food Safety Authority (NZFSA) released a risk management strategy for reducing incidence of poultry-associated foodborne campylobacteriosis.

New Zealand has a highly integrated, closed system of poultry production, with all poultry meat available for retail sale being of domestic origin. Processors of poultry meat control most aspects of production, processing, and distribution; 3 processing companies supply >90% of chicken meat consumed in New Zealand (2). As a result, interventions applied to the local poultry industry affect all domestically consumed poultry.

A marked decline in campylobacteriosis notifications was observed during 2007 and 2008 (17). We investigated this decline to assess whether it was causally related to the poultry-focused food safety interventions.

## Methods

### Descriptive Epidemiology

Historic notification and hospitalization data were used to calculate annual rates of campylobacteriosis in New Zealand during 1980–2009 for notifications and 1996–2009 for hospitalizations. A detailed descriptive analysis was then undertaken to examine the epidemiology of campylobacteriosis for the 12-year period 1997–2008 on the basis of notified and hospitalized cases.

Campylobacteriosis notification data are collated nationally by the Institute of Environmental Science and Research Ltd from notifications made by medical practitioners to their local public health service. During the study period, >96% of these notifications were culture-confirmed cases, with the remainder being epidemiologically linked to confirmed cases. Hospitalization data are collated by the Ministry of Health from information supplied by public hospitals. Analysis of hospitalized cases was based on patients with a principal diagnosis code for *Campylobacter* enteritis (International Classification of Diseases, 9th Revision, Clinical Modification, code 008.43, or International Classification of Diseases, 10th Revision, code A04.5). These data were further selected to exclude hospital transfers, readmissions within 30 days, and day cases (i.e., patients assessed in hospital for a short time but not requiring an overnight stay). Admissions to private hospitals were excluded because few patients with infectious diseases are admitted to such institutions and documentation is inconsistent.

In the detailed descriptive analysis, temporal trends in disease incidence and distribution were examined according to patient age, sex, socioeconomic status, ethnicity, urban versus rural dwelling, region (health board area), and season. Case-patients were assigned rurality and deprivation scores on the basis of their home domicile. For rurality assignment, we used a Statistics New Zealand classification system, which defines 7 grades of rurality on the basis of population size and employment address (18). Socioeconomic status was measured by deprivation scores assigned according to the New Zealand Deprivation Index, an area-based measure of socioeconomic position derived from the 5-year Census of Population and Dwellings (19).

The main descriptive analysis rates were calculated by using interpolated and extrapolated Census Usually Resident population data from 1996, 2001, and 2006. Rates for 2007 and 2008 (with 2007 being the transition year, on the basis of the gradual implementation of interventions) were compared with the average annual rates for 2 baseline periods (1997–2001 and 2002–2006). For the longer time-trend analysis, rates were calculated by using mid-year population estimates derived by Statistics New Zealand (20).

To examine the stability of the notification system for enteric diseases during the period of interest, we compared rates for campylobacteriosis notification and hospitalization with rates for 3 other notifiable enteric diseases (salmonellosis, yersiniosis, and cryptosporidiosis). Ethical approval for this study was obtained from the Multi-Region Ethics Committee, Wellington, New Zealand.

### Source Attribution

During March 2005–December 2008, *C. jejuni* isolates from human case-patients and environmental and food sources were collected in the Manawatu area and genotyped (sequence-typed) by using MLST (12,16). Food samples were collected from fresh meat (poultry, beef, lamb) in retail stores, and environmental water samples were collected from swimming locations in rivers. Sheep and cattle feces were sampled from farms adjacent to the catchments of these rivers.

Two models were used to apportion human cases to sources on the basis of sequence types: the modified Hald model and the Island model (12,15). The modified Hald model combines the prevalence of each *C. jejuni* sequence type among the sources with the observed number of human isolates of that type by using a Bayesian framework (15). This model includes source-specific and type-specific factors, and accounts for variation in the estimated prevalence. The source-specific factor gives a measure of the ability of a source to act as a vehicle for human infection, whereas the type-specific factor yields a measure of the ability of a particular sequence type to cause disease.

The Island model uses an evolutionary model to assign sequence types to a particular source “island” or population (12). Mutation, recombination, and migration rates for isolates within and between each island are estimated by using the source isolates, and the posterior distribution of these estimates are then used to infer the origin of human isolates (12,13). To account for variations in food-processing practices that may affect the likelihood of human infection from each food source, we further extended both models to examine whether changes had occurred over time in the relative contribution of different sources to human campylobacteriosis (dynamic modeling) (21).

### Key Informant Interviews and Policy Review

Key informants (n = 12), including industry and food safety experts, were interviewed to obtain information on interventions implemented to reduce *Campylobacter* spp. contamination in poultry. We used information from these interviews together with a review of policy documents from NZFSA and the poultry industry to formulate a summary of the interventions implemented from 2006 through 2008.

## Results

### Descriptive Epidemiology

**Figure 1:**
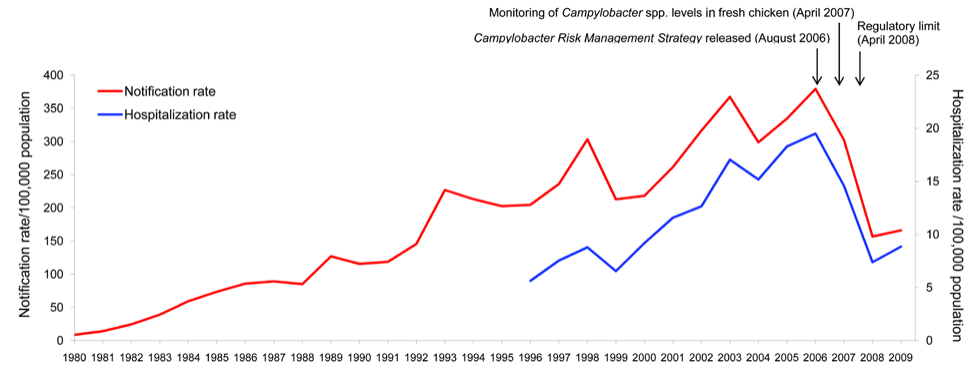
Campylobacteriosis notification rates per 100,000 population by year, 1980–2009, and hospitalization rates per 100,000 population by year, 1996–2009, New Zealand. Arrows indicate key interventions.

The time-trend analysis of annual notification and hospitalization rates demonstrates a steady rise and then a marked decline in the incidence of campylobacteriosis (Figure 1). In the detailed descriptive analysis covering 1997–2008, the 2008 annual rate for campylobacteriosis notifications was 161.5/100,000 population, representing a 54% decline compared with the average annual rate of 353.8 for 2002–2006 (Technical Appendix). The 2008 campylobacteriosis hospitalization rate of 7.6/100,000 population represented a 56% decline compared with the average annual rate for 2002–2006 of 17.3/100,000 population (Figure 1).

Statistically significant declines in notifications were evident across all analyzed population subgroups, although the magnitude of the declines varied (Technical Appendix). Similarly, significant decreases were seen for most subgroups for campylobacteriosis hospitalizations (results not shown).

**Figure 2:**
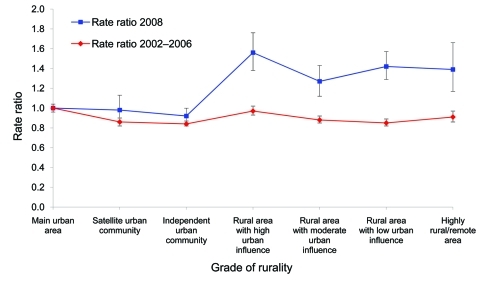
Rate ratios of campylobacteriosis notifications in New Zealand by grade of rurality for 2002–2006 and 2008. Main urban area was used as reference value for rate ratios. Error bars indicate 95% confidence intervals.

For the 2002–2006 period (before the decline), a trend for lower notification rates was shown among those residing in more rural areas compared with those living in main urban areas (rate ratios [RR] <1 where the reference is “main urban areas”) (Figure 2). In contrast, significantly higher notification rates were observed among those residing in more rural areas compared to those living in main urban areas in 2008 (Figure 2; Technical Appendix), indicating greater declines in incidence occurred in urban areas than in rural areas during 2007–2008.

The largest declines in campylobacteriosis notification rates between the average annual rate for 2002–2006 and the 2008 rate were seen in winter months (RR 0.38, 95% confidence interval [CI] 0.36–0.40), in urban populations (RR 0.42, 95% CI 0.41–0.43), in the age groups 20–29 years and 30–39 years (RR 0.40, 95% CIs 0.38–0.43 and 0.37–0.43, respectively) and in the Asian ethnic group (RR 0.26, 95% CI 0.22–0.31) (Technical Appendix).

Conversely, the smallest declines in notification rates comparing the 2008 rate with the average annual rate for 2002–2006 were seen in rural populations (RR 0.66, 95% CI 0.62–0.70), in the 0–4 and the >80 years-of-age groups (RR 0.63, 95% CI 0.59–0.67, and RR 0.61, 95% CI 0.53–0.70 respectively) and in Māori, the indigenous people of New Zealand (RR 0.49, 95% CI 0.44–0.55) (Technical Appendix).

**Figure 3:**
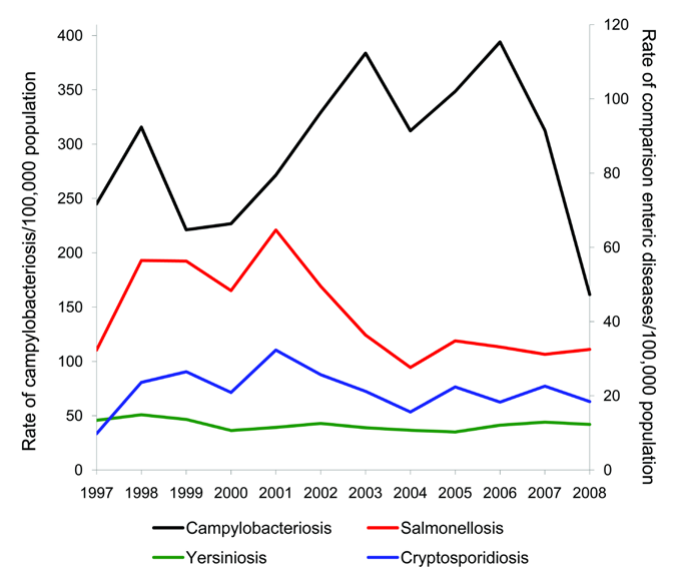
Annual campylobacteriosis notification rates per 100,000 population compared with annual notification rates per 100,000 population for salmonellosis, cryptosporidiosis, and yersiniosis, New Zealand, 1997–2008.

Figure 3 shows the temporal relationship between campylobacteriosis notification rates for 1997–2008 and 3 other notifiable enteric diseases. The marked decline in campylobacteriosis notifications during 2007–2008 is evident, while over this same period, salmonellosis, cryptosporidiosis, and yersiniosis rates remained relatively stable

### Source Attribution

**Figure 4:**
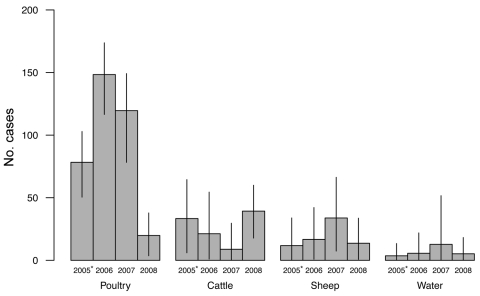
Number of cases attributed to source by year as determined by the modified Hald model in the Manawatu region of New Zealand. Error bars indicate 95% credible intervals. *2005 data are March through December only.

During the study period 2005–2008, 572 human *C. jejuni* isolates and 811 food and environmental isolates were collected (and had complete MLST profiles available). The estimated number of cases attributable to each source over time (based on the dynamic modified Hald model) is shown in Figure 4. These data show that compared with the baseline period (2005–2006), the number of cases in the Manawatu region attributed to poultry declined by 74% (95% credible interval 49%–94%) in 2008. No evidence was found for a decline in cases attributed to nonpoultry sources over the same period (p>0.5) (Figure 4). Similar results were obtained for the dynamic version of the Island model (results not shown).

### Summary of Interventions

Specific food safety and poultry industry interventions were implemented beginning in 2006, in line with NZFSA’s strategy for reducing the incidence of foodborne campylobacteriosis (Tabl[Table T1]e). From April 2007, poultry processors monitored and reported to the NZFSA-administered National Microbiological Database *Campylobacter* spp. prevalence in poultry flocks by using presence/absence cecal testing and *Campylobacter* spp. contamination levels in poultry carcass rinsates at the end of primary processing (Table).

**Table T1:** Key regulator and industry interventions and activities introduced in 2006–2008 to reduce poultry-associated foodborne campylobacteriosis, New Zealand*

Step	Initiative	Aim	Comments
Primary production	Development of voluntary Broiler Growing Biosecurity Manual by industry, building on existing industry biosecurity manuals and codes of practice	Identify effective on-farm biosecurity procedures in the New Zealand context; set industry best practice for on-farm biosecurity to help prevent *Campylobacter* spp. infection of flocks	Implemented in August 2007; developed by industry based on evaluation of existing on-farm biosecurity procedures and review of national and international best practice†
	Improvements in procedures for catching and transporting birds and for cleaning/drying of transport crates	Reduce possible cross infection between infected and non-infected birds during transport	
	Monitoring and reporting prevalence of *Campylobacter* spp. in cecal samples taken from birds from each growing shed each time birds are sent for processing	Determine the proportion of infected flocks; aid investigation of risk factors for flock infection; identify poor-performing farms	Implemented April 2007; reported to National Microbiological Database, administered by NZFSA‡
Processing	Monitoring and reporting enumerated levels of *Campylobacter* spp. from rinsates of bird carcasses exiting the immersion-chiller (at the end of primary processing)	Assess the effectiveness of risk mitigation strategies implemented on-farm and during processing in reducing *Campylobacter* spp. levels; inform development of national targets for *Campylobacter* spp. contamination at the end of primary processing	Implemented April 2007; reported to the National Microbiological Database, administered by NZFSA
	Industry exchange of information and implementation of improvements during primary processing (particularly immersion-chiller conditions)	Identify cost-effective processing interventions that reduce the levels of *Campylobacter* spp. on broilers at completion of primary processing; inform an updated industry Code of Practice for primary poultry processing	2006–2008
	Implementation of an updated industry Code of Practice for primary processing of poultry (slaughter and dressing)	Set industry best practice for primary processing based on knowledge gained from previous processing trials	Issued August 24, 2007; implemented March 2008§
	Mandatory targets for *Campylobacter* spp*.* contamination levels on poultry carcasses after primary processing	Enable regulatory action to occur if poultry processors exceed a certain level of *Campylobacter* spp*.* contamination on broiler carcasses at the end of primary processing (on exiting the immersion-chiller)	Implemented April 2008; reported to the National Microbiological Database administered by NZFSA
Retail	Voluntary use of leak-proof packaging	Reduce potential for cross-contamination from contaminated packaging in retail and home settings	Introduced for whole carcasses by most primary processors. Introduced for portion packs by some supermarkets
	Intermittent monitoring of *Campylobacter* spp*.* contamination of retail poultry	Assess *Campylobacter* spp. levels in retail packs purchased by consumers; inform interventions and code of practice for secondary processing	Reflects *Campylobacter* spp. levels at primary processing and subsequent changes due to secondary processing, storage, distribution, and processing/handling at the retail outlet
Consumer	Enhanced consumer education	Increase public awareness of food safety risk mitigation behaviors	Initially instigated in 1998 by NZFSA and the existing New Zealand Food Safety Partnership
Other	Enhanced human campylobacteriosis surveillance and source attribution research	Monitor source attribution of human campylobacteriosis to guide future interventions	Source attribution work is ongoing to monitor the proportion of human campylobacteriosis cases attributable to different sources and transmission pathways

*NZFSA, New Zealand Food Safety Authority. †www.pianz.org.nz/Documents/Version_1.pdf. ‡Mandatory cecal testing was discontinued July 2009. §www.foodsafety.govt.nz/elibrary/industry/Code_Practice-Zealand_Food.htm.

In April 2008, mandatory *Campylobacter* spp. performance targets were introduced based on enumerated levels of *Campylobacter* spp. contamination on poultry carcasses at the end of primary processing, with escalating regulatory responses if targets were not met (22). NZFSA has subsequently released an updated *Campylobacter* Risk Management Strategy (23).

Key informants noted that attention to detail with hygienic practices throughout production and primary processing and alterations to the immersion-chiller conditions were key areas in which improvements were made. Furthermore, the monitoring of *Campylobacter* spp. contamination levels in poultry carcass rinsates at the end of primary processing and setting mandatory *Campylobacter* spp. performance targets (rather than mandating specific interventions) were viewed by both industry and regulator informants as key facilitators of the strategy’s success.

## Discussion

New Zealand experienced a marked decline in campylobacteriosis incidence during 2007, with the 2008 notifications and hospitalization rates >50% lower than the averages for 2002–2006. This decline was sustained in 2009 (Figure 1). This decreased incidence implies 70,000 fewer community cases in New Zealand in 2008 compared with the peak in 2006, on the basis of the widely used multiplier of 7.6× the number of notified cases occurring in the community (24).

This reduction in incidence corresponds closely in time to the introduction of voluntary and regulatory interventions to reduce contamination of poultry with *Campylobacter* spp. Furthermore, patterns of the decline in disease incidence by population subgroup and area, along with the lack of plausible alternative explanations, suggest a causal effect from the poultry-focused interventions. The greater decline in campylobacteriosis in urban populations compared to the decline in rural populations (Figure 2) suggests that changes in foodborne transmission pathways were a key driver of the decline, compared with exposure pathways more likely to be encountered in rural settings (e.g., direct contact with contaminated environments or animals).

Source attribution modeling also provides supportive evidence that the decline in human campylobacteriosis can be largely attributed to a reduction in infection arising from poultry. The attribution study suggested a 74% decline in cases originating from poultry sources in 2008 compared with the baseline for 2005–2006. No statistically significant declines in attribution were found for any other sources (Figure 4).

It is difficult to attribute the decline in poultry-associated human disease to any single intervention, because a range of food safety and poultry industry interventions were implemented since 2006. However, key informants identified the monitoring and reporting of *Campylobacter* spp. enumeration levels on poultry carcasses at the end of primary processing as particularly important, as well as the setting of mandatory performance targets.

The fall in campylobacteriosis rates in New Zealand is unusual in terms of the size and speed of the decline, and the regulatory measures that were used. Internationally, a small number of countries have reported declines in campylobacteriosis incidence following the implementation of control strategies focusing on poultry (25–28). These countries have used various interventions, but a commonality has been strengthening on-farm biosecurity and monitoring the prevalence of *Campylobacter* spp.–positive flocks.

Although substantial evidence exists that poultry industry interventions contributed to the decline in campylobacteriosis incidence in New Zealand, several alternative explanations should be considered. These include the possibility of surveillance artifact, declining poultry consumption, declining disease associated with other foods or drinking water, effects of climate, and changes in consumer behavior.

Surveillance artifact is unlikely to have contributed significantly to the decline, however, given the magnitude of the reduction, the similarity of temporal trends in hospitalization and notification data (Figure 1), the decline occurring across all population subgroups, and the lack of similar declines for the comparison group of notifiable enteric diseases (Figure 3). Furthermore, the decline in campylobacteriosis in 2007 and 2008 was observed for all geographic areas (albeit to varying degrees), which suggests a change in a ubiquitous and common exposure. Salmonellosis rates may also have been expected to fall because of the potential concomitant effects of the interventions on *Salmonella* spp. contamination of poultry. However, the lack of decline in salmonellosis is not surprising in the New Zealand context because *Salmonella* spp. contamination levels were very low in poultry before the implementation of these interventions (29).

To assess the possible impact of poultry consumption on the decline in campylobacteriosis, we examined poultry production data. In New Zealand, poultry production approximates poultry consumption because of the closed nature of the production system. Over the period of the marked decline in campylobacteriosis incidence (2006–2008), fresh poultry production waned by only 5.8% (30). While this fall in production could have affected the incidence of poultry-associated foodborne campylobacteriosis, it is unlikely to be sufficient to explain the >50% drop in campylobacteriosis notifications occurring over this period.

Several foodborne pathways of campylobacteriosis (other than poultry) have been identified, including red meat and raw milk consumption (9,31). The contribution of these pathways to sporadic campylobacteriosis in New Zealand has been estimated to be markedly less than that of poultry (9,12). The magnitude of the decrease seen in 2008 is such that even if the contributions from food sources other than poultry had been eliminated in their entirety, they likely could not account for the observed decline in campylobacteriosis.

Contaminated water and other environmental sources have been implicated as a transmission pathway of human campylobacteriosis (32,33). Although water is found to be contaminated with *Campylobacter* spp., molecular epidemiologic studies have shown a low similarity between these genotypes and those found in human case-patients, suggesting that the strains detected in water are relatively apathogenic or that humans have limited exposure to them (12). Furthermore, a high proportion of New Zealanders receive treated community water supplies, with only small gradual increases in the proportion receiving water that meets microbiological quality criteria (34).

Changes in consumer behavior (e.g., hygiene, food preparation, eating out) could have plausibly contributed to the decline. However, challenges in altering consumer behavior have been acknowledged (35), and, given the rapidity of the decline in incidence, it is unlikely a sudden, marked change in consumer behavior could have been a key driver of the decline.

The effect of climate was considered as a possible driver of the decline. Despite the seasonal pattern observed for campylobacteriosis, the main drivers of the association between climate and campylobacteriosis remain elusive (36). However, the rapidity of the fall in incidence suggests that global climate change factors are unlikely to be key drivers.

A strength of this study is the multiple data sources that were accessed and analyzed, including source attribution techniques and key informant interviews. Nevertheless, a limitation of this study in determining the likely cause of the recent decline in campylobacteriosis is the descriptive nature of the epidemiologic analysis and the complex epidemiology of campylobacteriosis, which means that not all factors that might influence the disease’s incidence were examined explicitly. Although validated by studies in 2 other regions, the source attribution analyses were from 1 sentinel site only, and this work also has its own limitations (12,13,15). A further weakness is that details of specific industry-level interventions to reduce poultry contamination are not in the public domain, and therefore cannot be examined in detail. We were also unable to examine in detail data on *Campylobacter* spp. contamination levels in poultry. However, summary microbiological data on *Campylobacter* spp. contamination levels from the national database for 2007 and 2008 as published in the updated *Campylobacter* Risk Management Strategy 2010–2013 (23) support a reduction in *Campylobacter* spp. prevalence and counts on poultry over the period of the decline.

Rates of campylobacteriosis have shown marked annual variations in the past, so it will be important to assess medium- to long-term trends in disease and its attribution to assess the effects of NZFSA’s strategy. Notification and hospitalization data for 2009 indicate that the decline in incidence seen in 2008 has been largely sustained (Figure 1). Despite the 2009 rates being slightly higher than those of 2008, they still represent a substantial decline compared with the average for 2002–2006 (48% for notifications and 50% for hospitalizations).

Although there are costs associated with implementing industry regulation, these are likely to be offset by both the direct and indirect savings from reduced disease effects and lost productivity, conservatively estimated to have cost NZ$600 per campylobacteriosis case in 2005 (37). Given an estimated 70,000 fewer cases of campylobacteriosis in the community in 2008 than in 2006, this decline represents notable savings to New Zealand society. While progress has been made in responding to New Zealand’s campylobacteriosis epidemic, the costs and effects are still significant. As such, further research (including evaluating additional interventions) is desirable from a public health perspective to enable continued reductions of the still high burden posed by campylobacteriosis.

The findings of this study provide evidence of a successful population-level food safety response to a serious public health issue. New Zealand has experienced a prolonged national epidemic of campylobacteriosis. Fresh poultry was implicated as the dominant source, and a range of voluntary and regulatory interventions were introduced to reduce *Campylobacter* contamination of poultry. The apparent success of these interventions demonstrates approaches other countries could consider for controlling infectious disease epidemics linked to specific food sources. This example highlights the importance of integrated public health surveillance that includes upstream hazards as well as disease (38). Finally, the success of the response shows the value of collaboration between industry, food safety regulators, and public health researchers in addressing important food safety issues.

## Supplementary Material

Technical Appendix Campylobacteriosis notification counts and rates.
