# Supplementary material for: Marked Campylobacteriosis Decline after Interventions Aimed at Poultry, New Zealand
Source: Emerg Infect Dis. 2011 Jun;17(6):1007–15. doi: 10.3201/eid1706.101272 (PMC3358198; doi:10.3201/eid1706.101272)
Supplement: Technical Appendix — Campylobacteriosis notification counts and rates. [file 10-1272-Techapp_6p.pdf]

Article DOI: 10.3201/eid1706.101272

Marked Campylobacteriosis Decline after Interventions Aimed at Poultry, New Zealand

Technical Appendix

Technical Appendix Table 1. Campylobacteriosis notification counts and rates per 100,000 for 1997–2001 and 2002–2006 (average annual) and 2007 and 2008 (annual), by season, age group, sex, prioritized ethnicity, deprivation, urban-rural dwelling, and health board area

	No. notifications*				Rate† (95% CI)				
Category	1997–2001	2002–2006	2007	2008	1997–2001	2002–2006	2007	2008	RR (95% CI)‡
Season§									
Summer	3,005	4,280	4,395	2,242	81.5 (80.2–82.8)	109.4 (108.0–110.9)	107.6 (104.4–110.8)	54.1 (51.9–56.4)	0.49 (0.47–0.51)
Autumn	2,029	2,642	2,740	1,227	55.0 (53.9–56.1)	67.5 (66.4–68.7)	67.1 (64.6–69.6)	29.6 (28.0–31.3)	0.44 (0.42–0.47)
Winter	1,797	3,048	2,563	1,229	48.7 (47.7–49.7)	77.9 (76.7–79.2)	62.7 (60.3–65.2)	29.7 (28.0–31.4)	0.38 (0.36–0.40)
Spring	2,613	3,871	3,080	1,995	70.8 (69.6–72.1)	99.0 (97.6–100.4)	75.4 (72.8–78.1)	48.1 (46.1–50.3)	0.49 (0.47–0.51)
Age group, y									
0–4	1,314	1,446	1,236	926	478.9 (467.5–490.6)	529.0 (516.9–541.3)	447.9 (423.6–473.6)	334.6 (313.7–356.8)	0.63 (0.59–0.67)
5–9	477	650	604	327	166.2 (159.7–173.1)	227.0 (219.3–234.9)	210.8 (194.6–228.3)	114.1 (102.4–127.2)	0.50 (0.45–0.56)
10–14	384	598	577	271	137.2 (131.2–143.4)	199.4 (192.4–206.7)	186.7 (172.1–202.6)	86.8 (77.1–97.8)	0.44 (0.39–0.50)
15–19	612	991	941	467	231.5 (223.4–239.8)	346.1 (336.6–355.9)	306.3 (287.4–326.5)	148.6 (135.8–162.8)	0.43 (0.39–0.47)
20–29	2,055	2,515	2,180	1,060	402.9 (395.2–410.7)	500.3 (491.6–509.1)	420.2 (403.0–438.2)	202.2 (190.4–214.8)	0.40 (0.38–0.43)
30–39	1,517	2,055	1,591	814	262.7 (256.8–268.7)	355.8 (349.0–362.8)	275.1 (261.9–288.9)	140.7 (131.3–150.7)	0.40 (0.37–0.43)
40–49	1,135	1,798	1,678	814	217.9 (212.3–223.6)	310.4 (304.1–316.9)	270.2 (257.6–283.4)	128.2 (119.7–137.3)	0.41 (0.38–0.44)
50–59	890	1,609	1,537	734	228.8 (222.2–235.6)	350.5 (342.9–358.3)	307.5 (292.5–323.2)	143.0 (133.0–153.7)	0.41 (0.38–0.44)
60–69	521	1,078	1,203	643	188.3 (181.2–195.7)	347.9 (338.8–357.3)	356.7 (337.1–377.4)	185.6 (171.8–200.5)	0.53 (0.49–0.58)
70–79	363	681	766	398	176.1 (168.2–184.4)	315.4 (305.0–326.2)	349.4 (325.5–375.1)	180.7 (163.8–199.3)	0.57 (0.51–0.63)
≥80	140	310	342	213	135.1 (125.4–145.5)	255.4 (242.9–268.4)	257.6 (231.7–286.4)	156.0 (136.4–178.4)	0.61 (0.53–0.70)
Unknown	36	109	123	26	–	–	–	–	–
Sex¶									
M	4,938	7,309	6,791	3,712	268.9 (265.6–272.3)	380.3 (376.4–384.3)	337.9 (329.8–346.0)	183.6 (177.7–189.6)	0.48 (0.46–0.50)
F	4,386	6,268	5,722	2,888	230.6 (227.5–233.7)	311.4 (307.9–314.9)	271.0 (264.0–278.2)	136.7 (131.7–141.8)	0.44 (0.42–0.46)
Unknown	119	264	265	93	–	–	–	–	–

Prioritized ethnicity¶									
Māori	315	636	653	349	56.8 (53.5–60.5)	118.5 (113.8–123.6)	117.1 (107.2–128.8)	58.2 (51.8–66.4)	0.49 (0.44–0.55)
Pacific Peoples	109	160	136	67	55.0 (49.3–61.8)	73.3 (67.7–79.8)	65.8 (53.2–82.5)	28.4 (21.5–39.2)	0.39 (0.3–0.5)
Asian	233	414	366	127	111.5 (104.5–119.2)	137.1 (130.7–144)	104.5 (92.9–118.5)	35.8 (29.0–44.9)	0.26 (0.22–0.31)
European / Other	6,813	9,507	8,575	3,600	264.4 (261.6–267.3)	360.0 (356.8–363.3)	316.1 (309.3–323)	134.1 (129.7–138.7)	0.37 (0.36–0.38)
Unknown	1,973	3,124	3,048	2,550	–	–	–	–	–
Deprivation quintile#									
Deciles 1–2	2,140	3,308	2,887	1,592	280.9 (275.7–286.3)	412.6 (406.4–419.0)	344.7 (332.4–357.5)	187.4 (178.4–196.9)	0.45 (0.43–0.47)
Deciles 3–4	1,732	2,851	2,597	1,420	233.2 (228.3–238.1)	361.2 (355.3–367.2)	316.0 (304.1–328.4)	170.5 (161.9–179.7)	0.47 (0.45–0.50)
Deciles 5–6	1,599	2,540	2,458	1,230	219.7 (214.9–224.6)	328.2 (322.5–333.9)	304.0 (292.2–316.2)	149.9 (141.8–158.6)	0.46 (0.43–0.49)
Deciles 7–8	1,361	2,197	2,044	1,115	188.8 (184.3–193.3)	286.8 (281.4–292.2)	254.2 (243.4–265.5)	136.5 (128.7–144.8)	0.48 (0.45–0.51)
Deciles 9–10	999	1,556	1,413	810	136.7 (133.0–140.6)	200.7 (196.3–205.2)	174.5 (165.7–183.9)	98.7 (92.1–105.7)	0.49 (0.46–0.53)
Unknown	1,614	1,389	1,379	526	–	–	–	–	–
Urban–rural status¶**									
Urban total	7,689	11,575	10,270	5,188	239.6 (237.2–242.0)	340.6 (337.8–343.4)	287.9 (282.3–293.5)	144.5 (140.5–148.5)	0.42 (0.41–0.43)
Rural total	1,205	1,663	1,492	1,113	227.4 (221.6–233.3)	313.3 (306.5–320.4)	273.1 (258.9–288)	206.1 (193.7–219.1)	0.66 (0.62–0.70)
Urban <15 y	1,522	2,011	1,785	1,004	215.7 (210.9–220.6)	276.8 (271.4–282.2)	241.4 (230.5–252.9)	135.0 (126.9–143.6)	0.49 (0.46–0.52)
Rural <15y	487	549	421	411	358.1 (344.1–372.6)	412.3 (397.2–428.1)	318.7 (289.6–350.6)	311.9 (283.2–343.6)	0.76 (0.69–0.84)
Urban ≥15y	6,138	9,468	8,382	4,166	250.1 (247.4–253.0)	359.6 (356.3–362.8)	302.0 (295.6–308.5)	147.6 (143.2–152.1)	0.41 (0.40–0.42)
Rural ≥15yr	715	1,108	1,062	697	181.8 (175.9–187.9)	265.0 (258.1–272.1)	242.4 (228.2–257.4)	156.7 (145.4–168.7)	0.59 (0.55–0.64)
Main urban	6,702	9,947	8,805	4,406	251.0 (248.3–253.7)	349.9 (346.8–353.0)	295 (288.8–301.3)	146.6 (142.2–151.0)	0.41 (0.40–0.42)
Satellite urban	210	369	338	187	184.0 (173.0–95.5)	300.0 (286.2–314.2)	265.8 (237.8–296.6)	143.4 (123.2–166.3)	0.48 (0.41–0.56)
Independent urban	777	1,260	1,127	595	182.3 (176.6–188.2)	295.3 (287.9–302.8)	253.5 (238.4–269.3)	134.6 (123.7–146.3)	0.46 (0.42–0.50)
Rural, high urban	220	386	388	274	211.1 (198.4–224.6)	339.3 (323.6–355.9)	317 (284.3–353.3)	228.5 (200.8–259.8)	0.64 (0.56–0.73)
Rural, inter. urban	288	446	422	284	204.3 (193.7–215.5)	309.5 (296.4–323.2)	279.7 (252.6–309.5)	185.8 (164.0–210.2)	0.60 (0.53–0.68)
Rural, low urban	476	626	526	427	220.4 (211.4–229.6)	298.7 (288.1–309.6)	248.5 (227.2–271.6)	208.2 (188.3–229.8)	0.67 (0.61–0.74)
Highly rural	220	205	156	128	319.8 (300.9–339.9)	320.0 (300.5–340.7)	253.0 (214.2–297.9)	203.5 (169.4–243.6)	0.63 (0.52–0.76)
Unknown	550	603	1,016	392	–	–	–	–	–

Health board area¶									
Northland	198	319	369	249	143.4 (134.5–152.8)	222.7 (211.7–234.2)	247.0 (221.5–274.9)	170.0 (149.0–193.5)	0.76 (0.66–0.87)
Waitemata	1,093	1,837	1,699	881	258.6 (251.7–265.5)	391.4 (383.3–399.6)	342.8 (326.6–359.8)	176.9 (165.4–189.1)	0.45 (0.42–0.48)
Auckland	997	1,528	1,381	699	263.0 (255.6–270.6)	374.6 (366–383.4)	328.9 (311.3–347.4)	166.3 (153.8–179.6)	0.44 (0.41–0.48)
Counties Manukau	735	1,237	1,118	624	200.1 (193.6–206.8)	300.8 (293.2–308.6)	250.3 (235.5–265.8)	139.1 (128.3–150.7)	0.46 (0.42–0.50)
Waikato	1,000	1,241	1,080	574	310.5 (301.9–319.2)	375.7 (366.4–385.2)	313.0 (294.5–332.4)	162.3 (149.1–176.4)	0.43 (0.39–0.47)
Lakes	176	336	288	154	179.9 (168.1–192.3)	347.4 (330.9–364.5)	293.5 (260.4–329.8)	154.3 (130.7–181.2)	0.44 (0.37–0.52)
Bay of Plenty	278	511	498	257	163.0 (154.4–171.8)	276.0 (265.2–287.1)	250.7 (228.5–274.5)	127.9 (112.4–145.2)	0.46 (0.4–0.52)
Tairawhiti	70	101	49	41	156.9 (140.8–174.4)	229.8 (210.1–251.0)	105.3 (77.5–140.9)	89.6 (64.0–122.9)	0.39 (0.28–0.54)
Taranaki	214	386	410	204	208.2 (195.9–221.1)	382.0 (365.1–399.7)	402.7 (364.2–444.3)	198.4 (171.7–228.3)	0.52 (0.45–0.60)
Hawke’s Bay	394	503	461	305	276.2 (264.1–288.7)	347.6 (334–361.6)	309.6 (281.6–339.8)	207.4 (184.5–232.7)	0.60 (0.53–0.68)
MidCentral	251	320	332	196	159.2 (150.5–168.3)	204.3 (194.4–214.7)	207.5 (185.6–231.4)	122.2 (105.6–140.9)	0.60 (0.52–0.70)
Whanganui	106	181	190	89	161.8 (148.2–176.3)	293.2 (274.2–313.3)	299.4 (257.1–347.3)	150.7 (120.2–187.1)	0.51 (0.41–0.63)
Capital and Coast	1,028	1,225	990	579	404.7 (393.5–416)	461.2 (449.6–473)	359.8 (337.6–383.3)	208.3 (191.6–226.3)	0.45 (0.41–0.49)
Hutt Valley	420	534	435	298	315.4 (302–329.2)	397.8 (382.8–413.3)	316.1 (287–347.5)	218.5 (194.3–244.9)	0.55 (0.49–0.62)
Wairarapa	70	97	69	49	188.4 (169–209.7)	260.5 (237.3–285.7)	178.2 (137.3–229.3)	140 (102.6–188.1)	0.54 (0.4–0.72)
Nelson Marlborough	152	309	409	198	129.4 (120.4–139)	247.7 (235.3–260.6)	318.5 (287.6–352)	150.1 (129.4–173.5)	0.61 (0.53–0.71)
West Coast	66	85	81	54	212.3 (189.7–236.9)	286.8 (259.8–316)	252.8 (198.4–318.7)	180.3 (134.6–237.8)	0.63 (0.47–0.84)
Canterbury	1,295	1,730	1,754	661	305.5 (298.1–313.1)	383.4 (375.3–391.6)	368.1 (350.9–385.9)	137.7 (127.4–148.8)	0.36 (0.33–0.39)
South Canterbury	177	276	220	145	348.4 (325.5–372.4)	547.2 (517.9–577.8)	432.3 (374.7–497.2)	294.7 (246.7–350.3)	0.54 (0.46–0.64)
Otago	456	694	613	273	261.5 (250.8–272.6)	392.1 (379–405.6)	333.7 (307.3–361.9)	149.8 (132.2–169.3)	0.38 (0.34–0.43)
Southland	270	391	332	163	257.1 (243.6–271.2)	375.7 (359.1–392.8)	311.8 (279.0–347.5)	154.3 (131.4–180.1)	0.41 (0.35–0.48)
Unknown	0	0	0	0	–	–	–	–	–

*Average annual number for 1997–2001 and 2002–2006 rounded to the nearest integer; annual counts for 2007 and 2008.
†Average annual rate per 100,000 population for 1997–2001 and 2002–2006 (based on the interpolated/extrapolated Census Usually Resident populations for 1996, 2001, and 2006 Censuses), and the annual rate per 100,000 population for 2007 and 2008, with 95% confidence interval (CI).
‡RR, rate ratio calculated comparing the 2008 annual rate to the 2002–2006 average annual rate.
§Per the accepted definitions in the New Zealand (Southern Hemisphere) context, Summer, December–February; Autumn, March–May; Winter, June–August; Spring, September–November.
¶Rates directly age-standardized to the age distribution of the New Zealand population at the 2001 Census with confidence intervals calculated according to the methods used for age-standardized data (*1*).
#1997–1998 cases linked to New Zealand Deprivation (NZDep) 1996 Index of Deprivation concordance, 1999–2003 cases linked to NZDep2001 Index of Deprivation concordance, and 2004–2008 cases linked to NZDep2006 Index of Deprivation concordance.
**1997–1998 cases linked to the 1996 Census urban–rural concordance, 1999–2003 cases linked to the 2001 Census urban-rural concordance, and 2004–2008 cases linked to the 2006 Census urban–rural concordance.

Technical Appendix Table 2. Campylobacteriosis notification counts and rates per 100,000 population for 2002–2006 (average annual) and 2008 (annual), by season, age group, sex, prioritized ethnicity, deprivation, urban-rural dwelling and health board area

	2002–2006			2008		
Category	No. notifications*	Rate† (95% CI)	RR‡ (95% CI)	No. notifications*	Rate† (95% CI)	RR‡ (95% CI)
Season§						
Summer	4,280	109.4 (108.0–110.9)	1.0	2,242	54.1 (51.9–56.4)	1.0
Autumn	2,642	67.5 (66.4–68.7)	0.62 (0.59–0.65)	1,227	29.6 (28.0–31.3)	0.55 (0.51–0.59)
Winter	3,048	77.9 (76.7–79.2)	0.71 (0.68–0.75)	1,229	29.7 (28.0–31.4)	0.55 (0.51–0.59)
Spring	3,871	99.0 (97.6–100.4)	0.90 (0.87–0.95)	1,995	48.1 (46.1–50.3)	0.89 (0.84–0.95)
Age group, y						
0–4	1,446	529.0 (516.9–541.3)	1.06 (0.99–1.13)	926	334.6 (313.7–356.8)	1.65 (1.51–1.81)
5–9	650	227.0 (219.3–234.9)	0.45 (0.42–0.49)	327	114.1 (102.4–127.2)	0.56 (0.50–0.64)
10–14	598	199.4 (192.4–206.7)	0.40 (0.36–0.44)	271	86.8 (77.1–97.8)	0.43 (0.38–0.49)
15–19	991	346.1 (336.6–355.9)	0.69 (0.64–0.74)	467	148.6 (135.8–162.8)	0.73 (0.66–0.82)
20–29	2,515	500.3 (491.6–509.1)	1.0	1,060	202.2 (190.4–214.8)	1.0
30–39	2,055	355.8 (349.0–362.8)	0.71 (0.67–0.75)	814	140.7 (131.3–150.7)	0.70 (0.63–0.76)
40–49	1,798	310.4 (304.1–316.9)	0.62 (0.58–0.66)	814	128.2 (119.7–137.3)	0.63 (0.58–0.69)
50–59	1,609	350.5 (342.9–358.3)	0.70 (0.66–0.75)	734	143.0 (133.0–153.7)	0.71 (0.64–0.78)
60–69	1,078	347.9 (338.8–357.3)	0.70 (0.65–0.75)	643	185.6 (171.8–200.5)	0.92 (0.83–1.01)
70–79	681	315.4 (305.0–326.2)	0.63 (0.58–0.69)	398	180.7 (163.8–199.3)	0.89 (0.80–1.00)
≥80	310	255.4 (242.9–268.4)	0.51 (0.45–0.57)	213	156.0 (136.4–178.4)	0.77 (0.67–0.89)
Unknown	109	–	–	26	–	–
Sex¶						
M	7,309	380.3 (376.4–384.3)	1.0	3,712	183.6 (177.7–189.6)	1.0
F	6,268	311.4 (307.9–314.9)	0.82 (0.81–0.83)	2,888	136.7 (131.7–141.8)	0.74 (0.71–0.78)
Unknown	264	–	–	93	–	–
Prioritized ethnicity¶						
Māori	636	118.5 (113.8–123.6)	0.33 (0.32–0.34)	349	58.2 (51.8–66.4)	0.43 (0.39–0.48)
Pacific peoples	160	73.3 (67.7–79.8)	0.20 (0.19–0.22)	67	28.4 (21.5–39.2)	0.21 (0.17–0.27)
Asian	414	137.1 (130.7–144.0)	0.38 (0.36–0.40)	127	35.8 (29.0–44.9)	0.27 (0.22–0.32)
European/other	9,507	360.0 (356.8–363.3)	1.0	3,600	134.1 (129.7–138.7)	1.0
Unknown	3,124	–	–	2,550	–	–
Deprivation quintile#						
Deciles 1–2	3,308	412.6 (406.4–419.0)	1.0	1,592	187.4 (178.4–196.9)	1.0
Deciles 3–4	2,851	361.2 (355.3–367.2)	0.88 (0.83–0.92)	1,420	170.5 (161.9–179.7)	0.91 (0.85–0.98)
Deciles 5–6	2,540	328.2 (322.5–333.9)	0.80 (0.76–0.84)	1,230	149.9 (141.8–158.6)	0.80 (0.74–0.86)
Deciles 7–8	2,197	286.8 (281.4–292.2)	0.70 (0.66–0.73)	1,115	136.5 (128.7–144.8)	0.73 (0.67–0.79)
Deciles 9–10	1,556	200.7 (196.3–205.2)	0.49 (0.46–0.52)	810	98.7 (92.1–105.7)	0.53 (0.48–0.57)
Unknown	1,389	–	–	526	–	–

Urban–rural status¶**						
Urban total	11,575	340.6 (337.8–343.4)	1.0	5,188	144.5 (140.5–148.5)	1.0
Rural total	1,663	313.3 (306.5–320.4)	0.92 (0.90–0.94)	1,113	206.1 (193.7–219.1)	1.43 (1.34–1.52)
Urban <15 y	2,011	276.8 (271.4–282.2)	1.0	1,004	135.0 (126.9–143.6)	1.0
Rural <15 y	549	412.3 (397.2–428.1)	1.49 (1.36–1.64)	411	311.9 (283.2–343.6)	2.31 (2.06–2.59)
Urban ≥15 y	9,468	359.6 (356.4–362.8)	1.0	4,166	147.6 (143.2–152.1)	1.0
Rural ≥15 y	1,108	265.0 (258.1–272.1)	0.74 (0.69–0.78)	697	156.7 (145.5–168.7)	1.06 (0.98–1.15)
Main urban	9,947	349.9 (346.8–353.0)	1.0	4,406	146.6 (142.2–151.0)	1.0
Satellite urban	369	300.0 (286.2–314.2)	0.86 (0.82–0.90)	187	143.4 (123.2–166.3)	0.98 (0.85–1.13)
Independent urban	1,260	295.3 (287.9–302.8)	0.84 (0.82–0.87)	595	134.6 (123.7–146.3)	0.92 (0.84–1.00)
Rural, high urban	386	339.3 (323.6–355.9)	0.97 (0.93–1.02)	274	228.5 (200.8–259.8)	1.56 (1.38–1.76)
Rural, intermediate	446	309.5 (296.4–323.2)	0.88 (0.85–0.92)	284	185.8 (164.0–210.2)	1.27 (1.12–1.43)
Rural, low urban	626	298.7 (288.1–309.6)	0.85 (0.82–0.89)	427	208.2 (188.3–229.8)	1.42 (1.29–1.57)
Highly rural	205	320 (300.5–340.7)	0.91 (0.86–0.97)	128	203.5 (169.4–243.6)	1.39 (1.17–1.66)
Unknown	603	–	–	392	–	–
Health board area¶						
Northland	319	222.7 (211.7–234.2)	0.59 (0.56–0.63)	249	170.0 (149.0–193.5)	1.02 (0.88–1.18)
Waitemata	1,837	391.4 (383.3–399.6)	1.04 (1.01–1.08)	881	176.9 (165.4–189.1)	1.06 (0.96–1.18)
Auckland	1,528	374.6 (366.0–383.4)	1.0	699	166.3 (153.8–179.6)	1.0
Counties Manukau	1,237	300.8 (293.2–308.6)	0.80 (0.78–0.83)	624	139.1 (128.3–150.7)	0.84 (0.75–0.93)
Waikato	1,241	375.7 (366.4–385.2)	1.00 (0.97–1.04)	574	162.3 (149.1–176.4)	0.98 (0.87–1.09)
Lakes	336	347.4 (330.9–364.5)	0.93 (0.88–0.98)	154	154.3 (130.7–181.2)	0.93 (0.78–1.11)
Bay of Plenty	511	276.0 (265.2–287.1)	0.74 (0.70–0.77)	257	127.9 (112.4–145.2)	0.77 (0.67–0.89)
Tairawhiti	101	229.8 (210.1–251.0)	0.61 (0.56–0.67)	41	89.6 (64.0–122.9)	0.54 (0.39–0.74)
Taranaki	386	382.0 (365.1–399.7)	1.02 (0.97–1.07)	204	198.4 (171.7–228.3)	1.19 (1.02–1.40)
Hawke's Bay	503	347.6 (334.0–361.6)	0.93 (0.89–0.97)	305	207.4 (184.5–232.7)	1.25 (1.09–1.43)
MidCentral	320	204.3 (194.4–214.7)	0.55 (0.52–0.58)	196	122.2 (105.6–140.9)	0.74 (0.63–0.86)
Whanganui	181	293.2 (274.2–313.3)	0.78 (0.73–0.84)	89	150.7 (120.2–187.1)	0.91 (0.73–1.13)
Capital and Coast	1,225	461.2 (449.6–473)	1.23 (1.19–1.27)	579	208.3 (191.6–226.3)	1.25 (1.12–1.40)
Hutt Valley	534	397.8 (382.8–413.3)	1.06 (1.02–1.11)	298	218.5 (194.3–244.9)	1.31 (1.15–1.50)
Wairarapa	97	260.5 (237.3–285.7)	0.70 (0.63–0.76)	49	140.0 (102.6–188.1)	0.84 (0.63–1.12)
Nelson Marlborough	309	247.7 (235.3–260.6)	0.66 (0.63–0.70)	198	150.1 (129.4–173.5)	0.90 (0.77–1.06)
West Coast	85	286.8 (259.8–316)	0.77 (0.69–0.84)	54	180.3 (134.6–237.8)	1.08 (0.82–1.43)
Canterbury	1,730	383.4 (375.3–391.6)	1.02 (0.99–1.06)	661	137.7 (127.4–148.8)	0.83 (0.74–0.92)
South Canterbury	276	547.2 (517.9–577.8)	1.46 (1.38–1.55)	145	294.7 (246.7–350.3)	1.77 (1.48–2.12)
Otago	694	392.1 (379.0–405.6)	1.05 (1.01–1.09)	273	149.8 (132.2–169.3)	0.90 (0.78–1.04)
Southland	391	375.7 (359.1–392.8)	1.00 (0.95–1.05)	163	154.3 (131.4–180.1)	0.93 (0.78–1.10)
Unknown	0	–		0	–	–
Totals	13,841	353.8 (351.2–356.5)	–	6,693	161.5 (157.7–165.4)	–

*Annual number for 2002–2006 rounded to the nearest integer; annual counts for 2008. 1.0, reference value.
†Average annual rate per 100,000 population for 2002–2006 (based on the interpolated/extrapolated Census Usually Resident populations for 2001 and 2006 Censuses), and the annual rate per 100,000 population for 2008, with 95% confidence interval (CI).
‡RR, rate ratio calculated comparing the specified comparison variable (reference variable in bold).

§Per the accepted definitions in the New Zealand (Southern Hemisphere) context, Summer, December–February; Autumn, March–May; Winter, June–August; Spring, September–November.
¶Rates directly age-standardized to the age-distribution of the New Zealand population at the 2001 Census with confidence intervals calculated according to the methods used for age-standardized data (*1*).
#1999–2003 cases linked to New Zealand Deprivation (NZDep) 2001 Index of Deprivation concordance, and 2004–2008 cases linked to NZDep 2006 Index of Deprivation concordance.
**1997–1998 cases linked to the 1996 Census urban–rural concordance, 1999–2003 cases linked to the 2001 Census urban-rural concordance, and 2004–2008 cases linked to the 2006 Census urban–rural concordance.

Reference

1. Bray F. Age-standardization. In: Parkin D, Whelan S, Ferlay J, Teppo L, Thomas D, editors. Cancer Incidence in Five Continents Vol. VIII. Lyon (France): International Agency for Research on Cancer; 2002. p. 87–8.
